# Reduced circulating progenitor cells in older adults with major depression: Evidence of accelerated biological aging

**DOI:** 10.1016/j.bbih.2026.101211

**Published:** 2026-03-03

**Authors:** Erica L. Vieira, Perla El-Ahmad, Yuliya Nikolova, Tarek Rajji, Breno S. Diniz

**Affiliations:** a Campbell Family Mental Health Research Institute, Centre for Addiction and Mental Health, Toronto, ON, Canada; b Department of Psychiatry, University of Toronto, Toronto, ON, Canada; c UConn Center on Aging & Department of Psychiatry, UConn School of Medicine, University of Connecticut Health Center, Farmington, CT, USA; d Department of Psychiatry, University of Texas Southwestern Medical Center, Dallas, TX, USA; e Department of Psychiatry, Washington University in St. Louis, St. Louis, MO, USA

**Keywords:** Late life depression, Circulating progenitor cells, Aging, EPC, vSELs

## Abstract

Major depression (or late-life depression [LLD]) is a prevalent and debilitating condition among older adults and is increasingly recognized as a contributor to accelerated biological aging. LLD has been associated with aging-related phenotypes, namely chronic inflammation and cellular senescence. However, stem and progenitor cell exhaustion, another pivotal hallmark of biological aging, remains understudied in this context. In this study, we investigated the absolute count and frequency of circulating progenitor cell populations in peripheral blood mononuclear cells (PBMC) of 38 older adults (LLD, n = 19; control, n = 19). CD34^+^ cell enrichment increased the detectable number of progenitor cells by 30- to 50-fold compared to unselected PBMCs. Individuals with LLD had significantly lower counts of endothelial progenitor cells (EPC; β = −0.95 [-1.57 to −0.33], p = 0.003), and very small embryonic-like stem cells (vSEL; β = −1.61 [-2.65 to −0.58], p = 0.002). Additionally, vSELs count negatively correlated with the severity of depressive symptoms (r = −0.34, p = 0.034). These findings suggest that LLD is associated with reduced circulating progenitor cells, supporting the hypothesis that depression contributes to accelerated biological aging in older adults.

## Highlights

• **Late-life depression is associated with reduced circulating progenitor cell populations, including EPCs and vSELs.**
• **CD34^+^ enrichment improved detection of rare progenitor cells by 30- to 50-fold over unselected PBMCs.**
• **VSEL counts negatively correlated with depressive symptom severity, linking progenitor cell loss to clinical outcomes.**
• **Findings support stem cell exhaustion as a potential biological aging mechanism in late-life depression**.

## 1 Introduction

Late-life depression (LLD) is a highly prevalent psychiatric disorder among older adults and represents a significant public health concern (Byers et al., 2010). It is associated with reduced quality of life, functional disability, increased medical comorbidities, and a higher risk of age-related diseases, including Alzheimer's disease and related dementia (Diniz et al., 2013; Onyeka et al., 2019). Emerging evidence suggests that LLD drives accelerated biological aging, potentially explaining its link to poor physical health outcomes and neurodegenerative conditions (Diniz et al., 2026; Diniz et al., 2026a, Diniz et al., 2026b).

Stem cell (SCs) exhaustion is one of the main hallmarks of biological aging (Lopez-Otin et al., 2023). It reflects the decline in regenerative capacity necessary for tissue maintenance and repair, and their function declines with aging (Rando et al., 2025). Studying SC populations in humans is challenging due to their low numbers, heterogeneous nature, and residing within protected niches such as the bone marrow (Brunet et al., 2023). SCs often give rise to progenitor cells (PCs), which are early-lineage cells with limited self-renewal capacity and more restricted differentiation potential (Seaberg and van der Kooy, 2003). Several PC types circulate in the peripheral blood at low levels and serve as markers of systemic regenerative capacity. These include endothelial progenitor cells (EPCs), hematopoietic progenitor cells (HPCs), and very small embryonic-like stem cells (vSEL). The EPCs are precursors to endothelial cells involved in angiogenesis and vascular repair; HPCs give rise to myeloid and lymphoid cells; and vSELs are thought to be pluripotent-like cells capable of differentiating into multiple cell types, including hematopoietic cells (Ratajczak et al., 2008; M. Pérez et al., 2018).

Prior studies have reported that individuals with major depression have reduced circulating levels of EPC, which may increase in response to treatment with selective serotonin reuptake inhibitors (SSRIs)(Dome et al., 2009; Lopez-Vilchez et al., 2016). In contrast, elevated vSEL counts have been reported in bipolar disorder (Reginia et al., 2020). However, to date, no study has investigated circulating EPC, HPC, and vSEL count in individuals with LLD. Given the growing evidence implicating biological aging processes in LLD (Lorenzo et al., 2023), evaluating the progenitor cell population may offer novel insights into the complex relationship between LLD and accelerated biological aging. Thus, we aimed to evaluate the levels of circulating EPCs, HPC, and vSEL in older adults with and without LLD. Our primary hypothesis was that individuals with LLD would exhibit significantly lower levels of PCs compared to non-depressed individuals.

## 2 Methods

### 2.1 Recruitment of participants and assessment

Thirty-eight participants (LLD, n = 19; controls, n = 19) were recruited at the Centre for Addiction and Mental Health (CAMH), Toronto, Canada. Participants with LLD were diagnosed according to the Diagnostic and Statistical Manual of Mental Disorders, 5th edition (DSM-V). Older adults without a history of major depressive episodes, other psychiatric disorders, or hazardous substance use were included as a control group. We used the Mini International Psychiatric interview (MINI) to confirm the diagnosis of a major depressive episode or exclude a lifetime psychiatric disorder history (controls). Participants with stable medical conditions, such as hypertension or diabetes, were included in the study. Exclusion criteria for all participants were a previous diagnosis of dementia or Montreal Cognitive Assessment (MOCA)(Nasreddine et al., 2005) scores below 23 points, presence of current unstable medical illness, including recent history of cancer (except non-melanoma skin cancer), and history of alcohol or recreational drug abuse. In addition, participants with LLD were not under antidepressant treatment for at least three months before the research assessment and blood draw. The participants with LLD were not under psychiatric care before recruitment to this study. At the time of recruitment, they were either self-referred to the study based on advertisements or referred by their PCP for psychiatric assessment due to depressive symptom complaints.

We collected demographic, anthropometric, and medical history data in the baseline assessment. The severity of depressive symptoms was evaluated using the Montgomery-Asberg Depression Rating Scale (MADRS)(Montgomery and Asberg, 1979). Cognitive performance was evaluated by the MOCA test. Medical comorbidity burden was evaluated using the Cumulative Illness Rating Scale-Geriatrics (CIRS-G)(Miller et al., 1992). The local ethics board approved this study. All participants provided written informed consent for participation in this study.

**PBMC purification**: Peripheral blood (10 mL) was collected in the morning from the ulnar vein of participants into EDTA-coated vacuum tubes by a qualified healthcare professional at the Clinical Laboratory at CAMH. All procedures adhered to safety and aseptic standards for handling sharps. PBMCs from older adults were isolated by density gradient centrifugation using Ficoll-Paque™ Plus (GE Healthcare).

Following isolation, PBMCs were resuspended in RPMI-1640 medium (Sigma-Aldrich, St. Louis, MO, USA) supplemented with 200 U/mL penicillin, 0.1 mg/mL streptomycin, 1 mM L-glutamine, and 10% heat-inactivated AB Rh^+^ human serum from male donors (IHS; Sigma-Aldrich). Cell viability was assessed using the trypan blue exclusion method. Fresh PBMCs (1 × 10^7^ cells/mL) were immediately used for CD34 positive isolation. Remaining PBMCs were cryopreserved at −80 °C for future experiments.

**CD34^+^ cell isolation:** Given the very low frequency of hematopoietic cells in the circulation, we first isolated CD34^+^ hematopoietic progenitor cells to enrich the sample for further flow cytometry analyses. These cells were isolated from PBMCs using the EasySep™ Human CD34 Positive Selection Kit II (StemCell Technologies) following the manufacturer's instructions. PBMCs were resuspended at a concentration of 1 × 10^8^ cells/mL in EasySep™ buffer (PBS containing 2% IHS and 1 mM EDTA), incubated with the CD34 selection cocktail for 15 min at room temperature, followed by magnetic nanoparticle addition and another 10-min incubation. The cell suspension was placed in an EasySep™ magnet for 5 min, retaining the magnet-bound CD34^+^ cells while the supernatant was discarded. This magnetic separation step was repeated to improve purity.

Isolated CD34^+^ cells were resuspended in binding buffer (PBS with 0.5% bovine serum albumin, BSA; Sigma-Aldrich), counted, and assessed for purity using flow cytometry with CD34-PE (clone 581; BD Biosciences). The typical CD34^+^ yield ranged from 0.1% to 0.5% of total PBMCs, with a post-selection purity >90% in most samples. Both the CD34^+^ and CD34^−^ fractions were analyzed by flow cytometry to confirm the efficiency of CD34^+^ cell isolation.

**Cell surface staining for flow cytometer analysis**: Fresh PBMCs, as well as CD34^+^ positive and negative fractions, were stained with fluorochrome-conjugated monoclonal antibodies: FITC, PE, Cy7, and APC-labeled antibodies targeting CD45, CD34, CD133, and relevant isotype controls (Thermo Fisher Scientific, Waltham, MA, USA; BD Pharmingen, San Diego, CA, USA). Staining was performed for 20 min at 4 °C in the dark. After staining, cells were fixed in 2% formaldehyde (Sigma-Aldrich) and acquired on a CytoFLEX S flow cytometer (Beckman Coulter, Brea, CA, USA).

Due to the low frequency of target populations, a minimum of 100,000 gated events was collected per sample. Data acquisition and analysis were performed using FlowJo® software (Tree Star, Ashland, OR, USA).

**Fig. 1:**
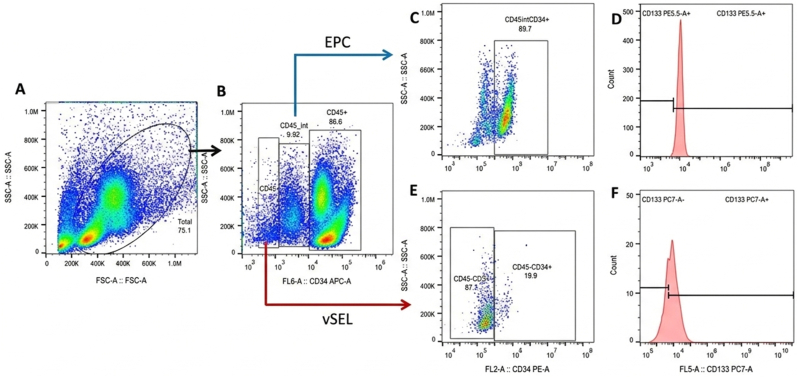
Flow cytometry gating strategy for identification of endothelial progenitor cells (EPCs) and very small embryonic-like stem/progenitor cells (VSELs). (A) Initial gating on lymphocyte and monocyte populations based on forward and side scatter profiles. (B) Cells were further gated by CD45 expression: CD45int cells were analyzed for EPCs, and CD45^−^ cells were analyzed for VSELs. (C) Within the CD45^int^ population, CD34^+^ cells were identified. (D) Histograms were generated to determine the frequency of CD45^int^CD34^+^ cells expressing CD133. (E) Within the CD45^−^ population, CD34^+^ cells were gated. (F) Histograms were used to assess the frequency of CD45^−^CD34^+^ cells co-expressing CD133.

**Flow cytometry data analysis:** Data analysis included gating on the lymphocyte and monocyte populations (Fig. 1A). Cells expressing intermediate levels of CD45 (CD45^int^) were analyzed for endothelial progenitor cells (EPCs), while CD45-negative (CD45^−^) cells were assessed for the presence of very small embryonic-like stem cells (VSELs) (Fig. 1B).

For EPC analysis, the CD45ˆint population was gated, followed by selection of CD34^+^ cells in a secondary dot plot (Fig. 1C). Histograms were then generated to assess the frequency of CD45^int^CD34^+^ cells expressing CD133 (Fig. 1D).

For VSEL-PCs identification, CD45^−^ cells were gated and analyzed for CD34 expression (Fig. 1E). Histograms were used to evaluate the frequency of CD34^+^ cells co-expressing CD133 (Fig. 1F). Gating thresholds were consistently established using isotype controls, fluorescence-minus-one (FMO) controls, and unstained negative populations.

### 2.2 Statistical analysis

The Student t-test was used to test for differences between LLD and controls for continuous variables. Categorical variables were compared using the chi-square test. Correlations between progenitor cell count and frequency and demographic or clinical measures were determined using Pearson correlation. We evaluate the association between diagnosis (LLD vs. Control) and the progenitor cell count using a negative binomial regression model, since these variables followed a negative binomial distribution. Additional covariates were included in the models as needed, based on statistically significant differences between groups. All statistical analyses were performed using Stata software version 17 for Windows (StataCorp, TX).

## 3 Results

Table 1 shows the demographic and clinical characteristics of the sample. As expected, individuals in the LLD group had significantly higher MADRS and CIRS-G scores, indicating a greater medical morbidity burden. There were no significant differences in sex distribution, age, cognitive performance, blood pressure, body mass index, and cardiovascular risk factors between groups.

**Table 1:** Socio-demographic, clinical, and biological characteristics of participants with and without late-life depression (LLD).

		Non-Depressed controls	LLD	Statistics	p-value
Age (years, mean ± SD)		70.2 ± 7.8	67.9 ± 5.9	t (37) = 1.05	0.3
Sex (%)	Men	52.6%	31.8%	χ^2^ (1) = 1.82	0.17
	Women	47.4%	68.2%		
MADRS (mean ± SD)		1.21 ± 1.51	16.0 ± 5.7	t (37) = 10.8	<0.001
MOCA (mean ± SD)		26.7 ± 2.1	26.6 ± 1.8	t (37) = 0.15	0.88
BMI (mean ± SD)		26.7 ± 3.7	28.11 ± 5.7	t (37) = 0.86	0.39
CIRS-G (mean ± SD)		6.9 ± 3.8	12.0 ± 9.1	t (37) = 2.6	0.012
Hypertension (%)	No	68.4%	72.7%	χ^2^ (1) = 0.09	0.76
	Yes	31.6%	27.3%		
Diabetes (%)	No	100.0%	90.9%	χ^2^ (1) = 1.81	0.18
	Yes	0.0%	9.1%		
Heart attack (%)	No	94.7%	100.0%		0.46
	Yes	5.3%	0.0%		
Heart failure∗ (%)	No	100.0%	95.5%		0.99
	Yes	0.0%	4.5%		
Angina (%)	No	100.0%	95.5%		0.99
	Yes	0.0%	4.5%		
HSC (cell count/mL) (mean count, 95%CI)		1192 (634 – 1750)	499 (260 – 738)	Wald χ^2^ (1) = 4.51	0.034
EPC (cell count/mL) (mean count, 95%CI)		1192 (634 – 1750)	499 (260 – 738)	Wald χ^2^ (1) = 6.87	0.009
vSEL (cell count/mL) (mean count, 95%CI)		37 (16 – 59)	9 (1 – 17)	Wald χ^2^ (1) = 17.39	<0.001

MOCA = Montreal Cognitive Assessment; MADRS = Montgomery–Åsberg Depression Rating Scale; BMI = Body Mass Index; EPC = Endothelial Progenitor Cells; vSEL = Very Small Embryonic-Like Stem Cells.

**Fig. 2:**
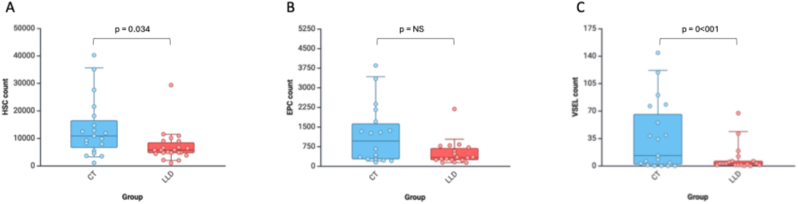
Box-plot graphs for distinct progenitor cell counts and percentage between LLD and Controls. A–C: absolute cell count per mL per 100,000 events. Data shown as median, interquartile range. HPC: Hematopoietic Stem Cell; EPC: Endothelial Progenitor Cell; VSEL: Very Small Embryonic-Like stem cell. The percentage of each progenitor cell type was calculated based on the fraction of CD34^+^ cells that were previously isolated. The absolute count of progenitor cells was calculated from the flow cytometry counts standardized per mL.

Individuals with LLD had significantly lower counts of HPC (Wald χ^2^2 (1) = 4.51, p = 0.034), EPC (Wald χ^2^ (1) = 6.87, p = 0.009), and vSELs (Wald χ^2^ (1) = 17.39, p < 0.001) compared to controls. This difference remained statistically significant after controlling for medical comorbidity burden for EPC (Wald χ^2^ (1) = 6.07, p = 0.014) and vSELs (Wald χ^2^ (1) = 19.38, p < 0.001), but not for HPC (Wald χ^2^ (1) = 2.12, p = 0.15) (Table 1 and Fig. 2). A lower vSEL count was significantly correlated with greater depressive symptoms (r = −0.34, p = 0.034). However, there was no significant correlation between HPC, EPC, vSEL count, or HPC, EPC, vSEL percentage and other demographic and clinical variables (Table 2).

**Table 2:** – Correlation between Endothelial Progenitor Cells, very Small Embryonic-Like Cells with clinical and psychiatric variables.

		EPC count	vSEL count
EPC count	Pearson Correlation	--	
vSEL count	Pearson Correlation	0.333	--
	Sig. (2-tailed)	0.054	
Age	Pearson Correlation	0.212	0.245
	Sig. (2-tailed)	0.215	0.139
MADRS	Pearson Correlation	−0.263	**−.343∗**
	Sig. (2-tailed)	0.122	**.035**
MOCA	Pearson Correlation	−0.039	0.307
	Sig. (2-tailed)	0.821	0.061
CIRS	Pearson Correlation	−0.047	−0.154
	Sig. (2-tailed)	0.790	0.371
Systolic blood pressure	Pearson Correlation	0.236	0.136
	Sig. (2-tailed)	0.201	0.450
Diastolic blood pressure	Pearson Correlation	0.143	0.078
	Sig. (2-tailed)	0.442	0.664
BMI	Pearson Correlation	0.087	−0.076
	Sig. (2-tailed)	0.637	0.668

MADRS = Montgomery–Åsberg Depression Rating Scale; MOCA = Montreal Cognitive Assessment; CIRS: Cumulative Illness Rating Scale; BMI = Body Mass Index; EPC = Endothelial Progenitor Cells; vSEL = Very Small Embryonic-Like Stem Cells.

## 4 Discussion

To our knowledge, this is the first study to evaluate the association between progenitor cell exhaustion and LLD. Our main findings were significantly lower counts of EPCs and vSEL in LLD that correlated with the severity of depressive symptoms. These findings provide preliminary support for the hypothesis that LLD is associated with accelerated aging effects in older adults (Lorenzo et al., 2023), and for identifying novel intervention targets (e.g., regenerative interventions aimed at restoring stem cell niches or improving their regenerative capacity) for the treatment of depressive symptoms and prevention of age-related adverse outcomes in this population.

Despite previous studies showing a decrease in progenitor cell populations, particularly EPCs, in depressed individuals (Dome et al., 2009; Chen et al., 2011), the underlying mechanisms for such associations remain poorly explored, especially in older adults. Cerebrovascular disease is a major contributor to the pathophysiology of LLD (Aizenstein et al., 2016), and major depression may exacerbate cardiovascular outcomes in older adults (Hare et al., 2014). Individuals with both cardiovascular and cerebrovascular disease have lower EPC count (Schmidt-Lucke et al., 2005). EPCs are recruited by cytokines and growth factors following vascular injury to the injury site, where they proliferate and differentiate, promoting angiogenesis (Pelliccia et al., 2022). It is possible that the continuous cycle of vascular injury and repair observed in LLD, exacerbated by the aging process, leads to the depletion and exhaustion of bone marrow stem cells, reflected by a decrease in EPC count.

The vSEL count was also reduced in LLD and significantly correlated with the severity of depressive symptoms. To our knowledge, this is the first study to investigate vSEL count in LLD. vSELs have a greater pluripotent potential and can be induced into different cell types, including other progenitor cells (e.g., HPC, EPCs), or terminally differentiated cells (e.g., neurons and cardiomyocytes)(Ratajczak et al., 2017). It has been suggested that vSELs are mobilized to the blood in response to stress, such as skin burns, myocardial infarction, and stroke, and are involved in tissue regeneration (Thetchinamoorthy et al., 2025). The decrease in vSELs count observed in individuals with LLD suggests that depression may negatively influence the ability of several tissues to respond to stressors and regenerate, making them more vulnerable to the negative effects of aging and susceptible to age-related illnesses and adverse health outcomes. On the other hand, reductions in the vSEL and EPC populations may be possible links to explain the higher incidence of cardio-metabolic and cerebrovascular disease, as well as neurodegenerative disorders, observed in individuals with LLD (Diniz et al., 2013).

Alternatively, our findings can be viewed from a broader perspective on how the mechanisms of biological aging and major depression interact (Lorenzo et al., 2023). For example, aging is characterized by low-grade, chronic, and systemic inflammation, which significantly contributes to morbidity and mortality in older adults (Ferrucci and Fabbri, 2018). Low-grade, chronic inflammation also plays a significant role in the pathophysiology of major depression, whereby individuals diagnosed with depression have an increase in pro-inflammatory markers such as interleukin-6 (IL-6), C-reactive protein (CRP), and tumor necrosis factor (TNF)-*α* (Drevets et al., 2022). The elevation of these pro-inflammatory markers can also play an inhibitory role in EPC differentiation and migration (Tousoulis et al., 2008), and, thus, can explain their lower counts in LLD. Another hallmark of aging is cellular senescence, which alters the secretome of cells, inducing the secretion of multiple factors known as the senescence-associated secretory phenotype (SASP)(Wang et al., 2024). Cellular senescence and related SASP components can change the stem cell niche microenvironment, leading to reduced proliferative and regenerative capacity of stem and progenitor cells (Bogeska et al., 2022; Moiseeva et al., 2023). Interestingly, we have reported that individuals with LLD exhibit a higher cellular senescence burden and increased SASP factors compared to non-depressed controls (Diniz et al., 2017; Seitz-Holland et al., 2023; Lorenzo et al., 2024). Therefore, it is plausible to hypothesize that the accumulation of pro-inflammatory and cellular senescence burden in LLD may also contribute to a decline in different progenitor cell populations as observed in our study.

Our results need to be interpreted in light of the study's limitations. Our sample size is relatively small, and the study participants were recruited from one psychiatric center, which may limit the generalizability of our findings. Also, the cross-sectional design of this study does not allow us to establish any causal relationships between progenitor cell count and LLD. Finally, our study was limited to only three progenitor cell types. Therefore, our findings should be replicated using a larger, more heterogeneous sample and a longitudinal design to obtain a more comprehensive understanding of the relationship between LLD and progenitor cell count.

In conclusion, participants with LLD have a lower count of EPCs and vSELs than non-depressed participants, and the vSELs count negatively correlated with more intense depressive symptoms. Our results reinforce recent findings suggesting a complex interaction between the mechanisms of LLD and biological aging processes.

## CRediT authorship contribution statement

**Erica L. Vieira:** Writing – original draft, Methodology, Formal analysis, Data curation, Conceptualization. **Perla El-Ahmad:** Writing – original draft, Formal analysis, Data curation, Conceptualization. **Yuliya Nikolova:** Writing – review & editing, Resources, Project administration. **Tarek Rajji:** Writing – review & editing, Supervision, Resources, Project administration. **Breno S. Diniz:** Writing – review & editing, Supervision, Project administration, Funding acquisition, Formal analysis, Conceptualization.

## Funding source

This study was funded by the NIMH grant 1R01MH115953.

## Declaration of competing interest

The authors have no competing interest related to this manuscript.

## Data Availability

Data will be made available on request.
