# Supplementary material for: Reduced circulating progenitor cells in older adults with major depression: Evidence of accelerated biological aging
Source: Brain Behav Immun Health. 2026 Mar 3;53:101211. doi: 10.1016/j.bbih.2026.101211 (PMC12993884; doi:10.1016/j.bbih.2026.101211)
Supplement: Multimedia component 1 [file mmc1.docx]

Supplemental Figure 1

**Figure 1S: Flow cytometry plots illustrating CD34⁺ cell isolation from PBMCs.**

Samples were stained with anti-CD34 and anti-CD45 antibodies. (A) Unenriched PBMCs showing baseline levels of CD34⁺ cells prior to magnetic selection. (B) Negative fraction after CD34⁺ cell isolation, showing cells expressing CD45 but lacking CD34 (CD45⁺CD34⁻). (C) Positive fraction enriched for CD34⁺ cells, showing co-expression of CD45 and CD34 (CD45⁺CD34⁺). Plots represent data from a single participant. SSC: Side Scatter; FSC: Forward Scatter.
